# Association of Irisin Serum Concentration and Muscle Strength in Normal-Weight and Overweight Young Women

**DOI:** 10.3389/fendo.2019.00621

**Published:** 2019-09-13

**Authors:** Ilse Yessabel Martínez Muñoz, Eneida del Socorro Camarillo Romero, Trinidad Correa Padilla, Jonnathan Guadalupe Santillán Benítez, María del Socorro Camarillo Romero, Laura Patricia Montenegro Morales, Gabriel Gerardo Huitrón Bravo, José de Jesús Garduño García

**Affiliations:** ^1^School of Medicine, Autonomous University of Mexico State, Toluca, Mexico; ^2^School of Chemistry, Autonomous University of Mexico State, Toluca, Mexico; ^3^Regional General Hospital No. 251, Mexican Institute of Social Security, Toluca, Mexico

**Keywords:** irisin, health-related to fitness, body composition, muscular strength, fat mass, hand grip strength

## Abstract

**Background:** Irisin is a muscle-contraction-induced myokine. In previous studies, it has been related to exercise type, fitness and physical activity; however, evidence is not consistent. Thus, the aim of this study was to research the association between health-related fitness and irisin in young women.

**Methods:** The study was designed as a prospective cross-sectional one. Young, healthy, nonsmoking women were enlisted. The sample comprised 40 overweight (OW) and 40 normal-weight (NW) individuals. The average age was 18.63 ± 0.63 and 18.78 ± 0.73 years, respectively. Components of health-related fitness, metabolic parameters, serum irisin and body composition were analyzed.

**Results:** Statistically significant differences were found in physical tests between NW and OW groups for one-leg standing, hand grip strength, vertical jump, modified push-up, fitness index and maximal oxygen uptake (VO_2MAX_). There were no differences in concentrations of serum irisin between the groups. We found a positive correlation between irisin and hand grip strength (*r* = 0.374, *p* = 0.023). In a multivariate analysis adjusted by body fat, a significant association between irisin and hand grip strength was observed in OW group (β = 0.380, *p* = 0.026); as well, a positive association between irisin and one-leg standing test in NW group (β = 0.311, *p* = 0.044) was found.

**Conclusions:** According to our findings, hand grip strength could be linked to irisin concentration in overweight young women.

## Introduction

In recent years, the global prevalence of overweight and obesity has increased (1). The age-standardized prevalence of obesity raised from 3.2% in 1975 to 10.8% in 2014 in men and from 6.4 to 14.9% in women (2). The association between excess weight and the development of chronic degenerative diseases is well-known (3). Therefore, a large part of the research has been addressed in order to search for preventive and therapeutic targets, focusing largely on adipose tissue and its different types (4).

In recent decades, skeletal muscle has been recognized as an endocrine organ, secretor of myokines, some induced by muscle contraction and proposed as intermediates between the absence of physical activity and the onset of chronic degenerative diseases related to obesity (5).

In 2012, irisin was first described as a hormone, product of cleave of a type 1 membrane protein encoded by the Fibronectin type 3 domain containing protein 5 (FNDC5), a gene capable of increasing energy expenditure, promoting weight loss and decreasing the resistance to insulin produced by diet (6) through mechanisms related to the browning of adipose and subcutaneous adipose tissue with a consequential increase in thermogenesis (7).

Irisin is a myokine induced by the contraction of skeletal muscle with implications in beneficial effects attributed to physical exercise (8). Cross-sectional and intervention studies have been carried out to link it to different types of physical exercise, components of fitness and physical activity, finding contradictory results (9–12).

Health-related physical fitness comprises aerobic fitness, musculoskeletal fitness, motor ability and body composition. Each component is measured using a different test. The monitoring of these components is relevant to avoid the risk of diseases associated with sedentism and also to promote the increase of physical capacity for daily activities (13). The aim of the present study was to associate irisin with health-related physical fitness components in young women.

## Materials and Methods

### Study and Subjects

This is a cross-sectional study carried out on young women students of *Universidad Autónoma del Estado de México* (UAEMex), aged between 18 and 20 years. Exclusion criteria were pregnancy, smoking, diabetes mellitus, cardiorespiratory diseases, thyroid disorders, hepatic failure, renal failure and inflammatory joint diseases or myopathies, as well as those who used drugs indicated for the diseases above. A total of 80 participants were included, 40 with normal weight (NW) and 40 with overweight (OW). BMI was 21.87 ± 1.55 and 27.01 ± 1.55 kg/m^2^, respectively.

This study was approved by the local Ethics and Research Committee (registration number 2016/06). All the procedures were performed according to relevant guidelines and regulations. Written informed consent was obtained from the participants.

### Measurements and Biochemical Parameters

We carried out a medical history, subsequently all measurements were performed after prior standardization, we measured blood pressure considering the average of two measurements with an interval of 2 min between each. Height was measured with a stadiometer seca® (Hamburg, Germany) and weight was measured by means of bioelectrical impedance Tanita® (Arlington, Ill, USA). BMI was calculated as weight (kg) divided by height squared (m^2^). Waist circumference was measured at the midpoint between the lowest rib and the iliac crest; while hip circumference, at the lateral position by measuring the circumference at the most prominent point. Body composition was evaluated by dual-energy X-ray absorptiometry (DXA) using a GE Lunar bone densitometer, GE Healthcare® (Little Chalfont, UK) wearing minimal clothing and no metallic objects.

Blood samples were taken between 08:00 and 09:00 h after fasting between 8 and 12 h. Plasma glucose was measured with the oxidized glucose method (Randox Laboratories Ltd, Antrim, UK); triglycerides with a colorimetric method following enzymatic hydrolysis performed with the lipase technique; total cholesterol was measured by cholesterol esterase; HDL cholesterol (HDLC) by the clearance method; uric acid was measured by the enzymatic colorimetric method. All biomedical assays were performed with a Selectra XL instrument (Randox Laboratories Ltd, Antrim, UK).

Serum irisin concentration was measured using the enzyme linked immunosorbent assay (ELISA) kit BioVendor (Brno. Czech Republic).

### Assessment of Health-Related Fitness

Health-related fitness was measured through the performance of tests corresponding to each type of fitness, as described below (14):

Motor fitness was assessed with the one-leg standing test, for which participants chose the leg they prefer to stand on, while the heel of the other leg was placed in the knee against the anterior site of the supporting leg, the thigh rotated outward and arms hung relaxed. The result was the longest time participants maintained the correct position twice (15).

Skeletal muscle fitness was assessed using hand grip strength, vertical jump and modified push-ups. Hand grip strength was measured with a dynamometer, Takei Scientific Instruments Co., Ltd. (Niigata-City, Japan) which was handled with the dominant hand keeping the arm straight and slightly away from the body. Participants squeezed firmly and gradually, until they reached the maximum strength, the best result of two performances was considered the score (16). Vertical jump consisted in jumping as high as possible after marking the height reached by the middle finger of the right hand of the participants standing with the arm raised and straight. The score was the maximum vertical difference in centimeters between standing height and that reached in the two jumps (17). Modified push-up test was performed face down; it consisted in placing the palms of hands at the beginning of the back and rising by flexing the arms so that the elbows remained completely straight. The result was the total number of correct push-ups performed over 40 s (18).

Cardiorespiratory fitness was assessed through a 2-kilometer walk test in an electric treadmill without elevation walking as fast as possible for the participant. The score of this test was determined through cardiorespiratory fitness (CF) and VO_2MAX_ following the formulas:

CF = 304–walking time (min) × 8.5 + walking time (s) × 0.14 + heart rate (beats/min) × 0.32 + BMI (kg/m^2^) × 1.1)–age (years) × 0.4.

VO_2MAX_ × (ml/min/kg) = 116.2–2.98 × walking time (min)−011 × heart rate (beats/min)−0.14 × age (years)−0.39 × BMI (kg/m^2^) (19, 20).

### Data Analysis

The descriptive analysis was expressed using means and standard deviations. Shapiro-Wilk test was performed to assess the distribution of variables. Differences between continuous variables were analyzed with Student's t test or Mann-Whitney U test, as appropriate. The analysis of continuous quantitative outcome variables was performed using Pearson correlation or Spearman's, as appropriate. Multivariate linear regression models were calculated adjusted for confounder variables. Variables were logarithmically transformed to fit in the model. Statistical analyses were run using Statistical software for Social Sciences (IBM SPSS Statistics for Windows, Version 22.0 Armonk, NY: IBM Corp).

## Results

Baseline subject characteristics are summarized in Table 1. Age, systolic blood pressure, diastolic blood pressure were similar between the groups. Waist circumference, hip circumference, percentage of total body fat and muscle mass were higher in OW group. Glucose, total cholesterol, LDL cholesterol, triglycerides and uric acid were also different between the groups.

**Table 1:** Baseline subject characteristics.

	**Normal weight**	**Overweight**	***p***
	***n* = 40**	***n* = 40**	
Age (years)	18.63 ± 0.63	18.78 ± 0.73	0.329
Body weight (kg)	55.20 ± 4.88	67.10 ± 6.92	0.001^*^
Height (cm)	158.93 ± 5.62	157.80 ± 5.88	0.389
BMI (kg/m^2^)	21.87 ± 1.55	27.01 ± 1.55	0.001^*^
Waist circumference (cm)	76.21 ± 5.34	86.53 ± 7.13	0.001^*^
Hip circumference (cm)	92.89 ± 3.05	100.60 ± 5.78	0.001^*^
Body fat (%)	36.09 ± 3.75	42.33 ± 3.31	0.001^*^
Muscle mass (kg)	33.44 ± 2.97	36.88 ± 3.28	0.001^*^
Systolic blood pressure (mmHg)	95.80 ± 8.76	98.89 ± 8.72	0.119
Diastolic blood pressure (mmHg)	66.53 ± 4.91	68.04 ± 3.92	0.132
Glucose (mg/dL)	89.90 ± 11.01	96.60 ± 10.67	0.007^*^
Total cholesterol (mg/dL)	158.63 ± 29.66	188.48 ± 35.41	0.001^*^
HDLC(mg/dL)	38.08 ± 3.81	38.35 ± 3.57	0.745
LDLC (mg/dL)	99.65 ± 25.26	118.70 ± 24.87	0.002^*^
Triglycerides (mg/dL)	98.61 ± 52.29	137.44 ± 62.37	0.003^*^
Uric acid (mg/dL)	3.74 ± 0.79	4.38 ± 1.21	0.006^*^
Irisin (ng/ml)	108.51 ± 70.08	126.63 ± 63.24	0.250

Data are presented as Mean ± SD.

* *p < 0.05 was considered statistically significant. BMI, Body mass index; HDLC, high-density lipoprotein cholesterol; LDLC, high-density lipoprotein cholesterol*.

**Figure 1:**
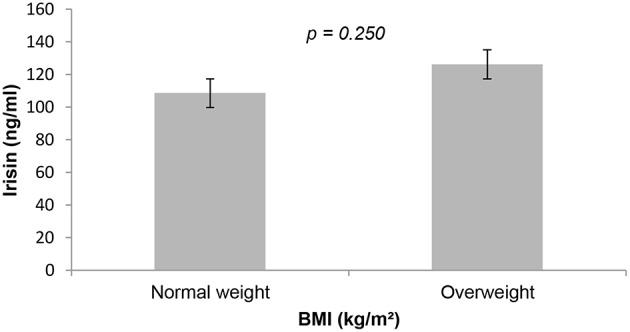
Irisin concentration in normal and overweight participants. *p* < 0.05 was considered as statistically significant. BMI, body mass index.

Health-related fitness was assessed through physical tests in both groups. NW group showed better performances in one-leg standing, vertical jump, modified push-ups, cardiorespiratory fitness and VO_2MAX_. On the other hand, OW group had a better performance in hand grip strength (Table 2). The overweight group had higher irisin concentrations compared with NW group, though there were no statistically significant differences (Figure 1).

**Table 2:** Results of physical tests.

	**Normal weight**	**Overweight**	***p***
	***n* = 40**	***n* = 40**	
One-leg standing (seconds)	49.38 ± 16.09	38.45 ± 18.95	0.007^*^
Hand grip strength (kg)	23.23 ± 3.69	25.81 ± 4.50	0.007^*^
Vertical jump (cm)	24.56 ± 4.75	21.66 ± 4.36	0.006^*^
Modified push-ups (number of correctly performed)	15.50 ± 5.84	12.80 ± 5.88	0.043^*^
Cardiorespiratory fitness	52.78 ± 17.82	39.11 ± 15.08	0.001^*^
VO_2MAX_(ml/min/kg)	28.10 ± 6.91	22.73 ± 7.04	0.001^*^

Data are presented as Mean ± SD.

* *p < 0.05 was considered statistically significant. VO_2MAX_: maximal oxygen uptake*.

**Figure 2:**
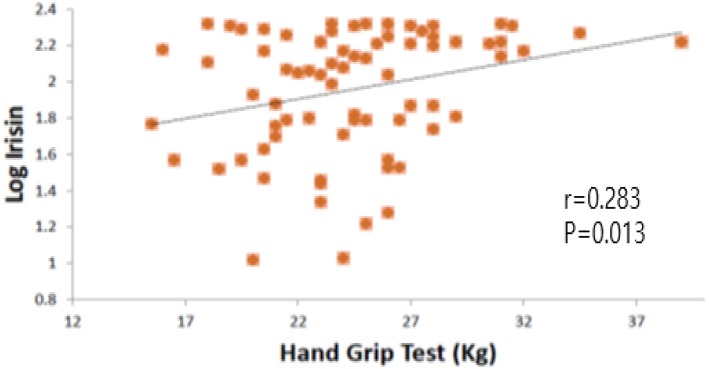
Log irisin and hand grip test correlation. *p* < 0.05 was considered statistically significant.

The correlation between health-related fitness tests and irisin concentration was calculated for the total population and for both groups. A statistically significant positive correlation was found between hand grip strength and irisin concentration in the total population (Figure 2). This correlation remains in OW group (*r* = 0.374, *p* = 0.023); however, NW group did not show any statistical significance (*r* = 0.129, *p* = 0.433). Multivariate linear regression models, adjusted for total fat, were produced; we found association between hand grip strength and irisin concentration in the total population and in OW group (Table 3).

**Table 3:** Multivariate linear regression models adjusted by fat mass between irisin concentration and health-related physical tests.

	**Total subjects**		**Normal weight**		**Overweight**	
	**β**	***p***	**β**	***p***	**β**	***p***
One leg standing	0.197	0.089	0.311	0.044	0.059	0.736
Hand grip strength	0.265	0.019	0.196	0.236	0.380	0.026
Vertical jump	0.140	0.355	0.230	0.161	−0.043	0.810
Modified push-ups	0.175	0.134	0.198	0.211	0.114	0.525
Cardiorespiratory fitness	−0.066	0.578	−0.099	0.536	−0.085	0.630
VO_2MAX_	−0.038	0.748	−0.131	0.412	0.023	0.898

*p < 0.05 was considered statistically significant. VO_2MAX_: maximal oxygen uptake; BMI: body mass index.

## Discussion

The present study describes the relation between irisin levels and health-related fitness in young women and the possible effect of overweight. We found a statistically significant positive correlation between hand grip strength and irisin concentration.

Obesity and overweight represent the main risk factors for the development of cardio-metabolic diseases. According to epidemiological studies, obesity and overweight incidence have grown as of the early eighties (21). In our study, we did not include diabetic patients, though metabolic risk factors such as lipid profile and fasting blood glucose seem to be different between NW and OW subjects. When we compared physical performance tests, cardiorespiratory, motor and some tests related to muscular fitness decreased in OW. Highlighting the effect of reduction of health-related fitness with body weight excess (22). Poor cardiorespiratory fitness has been considered an important cardiovascular risk factor and also a mortality predictor (23, 24). Shazia et al. (25) described the influence of excess body fat on aerobic fitness in young women.

In our results, irisin concentration was higher in OW group; however, it was not statistically significant. Previous studies have suggested that serum irisin concentration is higher in obese and overweight subjects compared with normal-weight subjects (26). These findings can be attributed to possible irisin resistance in presence of overweight. Park et al. (27) postulate that higher irisin concentrations in obese and overweight subjects could be related to a greater amount of fat and lean mass, and also to a possible compensatory role by irisin. Fukushima et al. (28) considered adipose tissue an influential factor in irisin secretion, especially in states of excess body fat. Previous studies have associated irisin concentration with cardiovascular fitness (29). In our study, we did not find a significant correlation between irisin and cardiorespiratory condition index or VO_2MAX_. It is not still clear if fasting irisin may have a correlation with cardiorespiratory condition or if it changes in response to intense exercising (30). In like manner, no statistical significance was found between irisin and vertical jump or modified push-ups. Hecksteden et al. (31) reported lack of association between irisin concentration and physical fitness after muscle and aerobic endurance training in adults.

We did not find any statistically significant correlation between physical tests and irisin. Although we did not find any statistically significant correlation between other physical tests and irisin, a positive correlation has been found between hand grip strength and irisin. Hand grip strength is considered a fast and simple test, proposed to be an indirect marker of muscle strength (16); A poor hand grip performance has been reported as a predictor for further development of type 2 diabetes mellitus (32). Therefore, hand grip strength may be an indirect marker of irisin. This concurs with a previous paper by Chang, who found a positive correlation between irisin and hand grip (33).

Since adipose tissue may influence the secretion of irisin (34), we adjusted irisin levels for body fat, and its association with hand grip strength remained significant after this adjustment. It should be mentioned that OW group also showed significantly higher amounts of muscle mass. The reason why statistical correlation between hand grip strength and irisin levels was found in the OW but not in the NW group may be due to higher muscle mass typically found in OW, compared to NW. Our findings are consistent with Kim et al. (35), who reported a statistically significant positive association between hand grip strength and irisin concentration in women after performing a resistance training program.

A possible association between one-leg standing test and irisin concentration was found only in NW group. One-leg standing is a useful test to identify bone deterioration and a decreased ability to perform this test is associated with increased risk of fractures (36). In our study, we included young, healthy women without bone-fracture risk factors; however, a relation between the osseous system and skeletal muscle, where irisin exercises endocrine functions on osteoblasts (37), has been mechanically and biochemically studied. *In vitro* and *in vivo* studies suggest that irisin stimulates osteoblasts to promote the formation of new bone tissue and improves strength and bone mass; though, studies in humans under different conditions are inconclusive (38).

The present study was limited by lack of comparability with a group of men; thereby, it was not possible to discuss the influence of gender on irisin and on the components of health-related fitness. Our sample included normal-weight and overweight women, it is suggested performing studies with higher BMI that allow observing the effect of fat percentage on irisin correlation and on components of health-related fitness. On the other hand, it is recommended carrying out intervention studies aimed at improving each of the components of health-related fitness that allow better analyzing the impact of each component on irisin concentration.

To sum up, irisin was not correlated with cardiorespiratory fitness test and its indexes such as VO_2MAX_ and cardiorespiratory fitness index. In the same way, there was no correlation between irisin and vertical jump and modified push-ups. However, we found an association between hand grip strength and irisin in overweight young women. In the normal weight group, one-leg standing test was associated with irisin concentration.

## Data Availability

Primary data is available from the authors.

## Ethics Statement

The authors certify that they complied with the ethical guidelines for authorship and publishing. The protocol was accepted by the local IRB and all the participants signed an informed consent letter.

## Author Contributions

IM and EC executed the research procedures, sample collection, laboratory analyses and data interpretation, designed the study, clinical management and laboratory analyses, interpreted data, contributed to the discussion, and reviewed and edited the manuscript. TC executed research procedures, sample collection, laboratory analyses, contributed to the discussion, and reviewed and edited the manuscript. JS executed laboratory analyses, contributed to the discussion, and reviewed and edited the manuscript. MC executed sample collection, laboratory analyses, contributed to the discussion, and reviewed and edited the manuscript. LM sample collection, laboratory analyses, contributed to the discussion, and reviewed and edited the manuscript. GH interpreted data, contributed to the discussion, and reviewed and edited the manuscript. JG executed the research procedures, sample collection and data interpretation, designed the study, clinical management, and laboratory analyses, interpreted data, contributed to the discussion, and reviewed and edited the manuscript. All the authors read and approved the final manuscript.

### Conflict of Interest Statement

The authors declare that the research was conducted in the absence of any commercial or financial relationships that could be construed as a potential conflict of interest.
